# Fatal Edwardsiella tarda Sepsis With Acute Enlargement of Multiple Abdominal Organs on Noncontrast Computed Tomography

**DOI:** 10.7759/cureus.117842

**Published:** 2026-10-13

**Authors:** Ako Negishi, Ryo Kurokawa, Yuko Sato, Mariko Kurokawa, Takashi Maki, Shota Fujisawa, Tomoki Wada, Osamu Abe

**Affiliations:** 1 Radiology, The University of Tokyo, Tokyo, JPN; 2 Emergency and Critical Care Medicine, The University of Tokyo, Tokyo, JPN; 3 Radiology, Graduate School of Medicine, The University of Tokyo, Tokyo, JPN

**Keywords:** computed tomography of the abdomen, edwardsiella tarda, emergency medicine, infectious disease medicine, radiology

## Abstract

*Edwardsiella tarda* (*E. tarda*) is a Gram-negative bacillus found in freshwater and brackish environments. Human infection is relatively uncommon and most frequently presents as gastroenteritis, although invasive infections may occur. We report a fatal case of *E. tarda* sepsis with the acute enlargement of multiple abdominal organs on computed tomography (CT). A 63-year-old woman with pancreatogenic diabetes mellitus and a history of surgery for malignancies presented with abdominal pain, vomiting, diarrhea, and headache. She developed severe lactic acidosis, disseminated intravascular coagulation, and multiorgan dysfunction. Noncontrast CT demonstrated the enlargement of both kidneys and adrenal glands, as well as the liver, compared with CT images obtained approximately six months earlier; given normal renal function eight days before admission and the fulminant course, these changes were highly likely to have developed acutely. The kidneys were diffusely enlarged, with increased attenuation of the perirenal and renal sinus fat. No definite abscess, gas within the organs, or focal fluid collection was identified. Despite intensive care, multiorgan failure progressed rapidly. Blood cultures yielded *E. tarda*, and subsequent history-taking revealed that the patient had consumed eel on the day of symptom onset. She died of multiorgan failure on day 9 after symptom onset. This case suggests that severe *E. tarda* sepsis may be associated with the diffuse enlargement of multiple abdominal organs even in the absence of a localized abscess or gas-forming lesion. When no apparent focal source of infection is identified despite a clinical presentation suggestive of severe infection, prompt blood culture testing and comparison with previous imaging studies are important, and a history of seafood or freshwater fish consumption, including eel, may provide a supportive clue.

## Introduction

*Edwardsiella tarda* (*E. tarda*) is a facultatively anaerobic Gram-negative bacillus found in freshwater and brackish environments, with fish, reptiles, and amphibians serving as its natural hosts. Human infection is relatively uncommon and most frequently presents as gastroenteritis; however, invasive infections, including bacteremia, biliary tract infection, liver abscess, soft tissue infection, and osteomyelitis, may occur [1-5]. Severe infection has been reported particularly in patients with underlying conditions such as liver disease, malignancy, diabetes mellitus, and immunosuppression [3,4].

Previously reported imaging findings of *E. tarda* infection have primarily involved localized sources of infection or suppurative lesions, including liver abscesses, pyelonephritis, psoas abscesses, and epidural abscesses. In a recent systematic review of 59 human cases, bilateral renal enlargement was described only in a patient with concomitant psoas abscesses [5]. To our knowledge, the simultaneous enlargement of both kidneys and adrenal glands, together with hepatic enlargement, in the absence of a discrete abscess or gas-forming lesion has not been well characterized in human *E. tarda* infection. Here, we report a fatal case of *E. tarda* bacteremia and sepsis in which noncontrast computed tomography (CT), compared with CT obtained approximately six months earlier, demonstrated the acute enlargement of both kidneys and adrenal glands, as well as the liver, followed by rapid progression to multiorgan failure. We present this case to describe the clinical and imaging features of fulminant *E. tarda* sepsis and to discuss possible mechanisms underlying the observed multiorgan enlargement.

## Case presentation

A 63-year-old woman had undergone surgery for intraductal papillary mucinous carcinoma of the pancreas 18 months previously and had been followed for postoperative pancreatogenic diabetes mellitus with a hemoglobin A1c level of 7.1%. She had also undergone surgery for early-stage colorectal cancer three years previously. At a routine outpatient visit eight days before admission, her serum creatinine level was 0.51 mg/dL, and her aspartate aminotransferase (AST) and alanine aminotransferase (ALT) levels were within the reference ranges. On the same day (day 1, defined as the day of symptom onset), she developed abdominal pain, vomiting, diarrhea, and headache. She remained bedridden at home and was transported to our hospital by ambulance on day 9.

On arrival, her blood pressure was 131/64 mmHg, heart rate was 168 beats/minute, respiratory rate was 25 breaths/minute, peripheral oxygen saturation was 97% on room air, and body temperature was 36.5°C. Although her blood pressure was initially maintained, marked tachycardia and tachypnea were present. The laboratory findings on admission are summarized in Table 1. Arterial blood gas analysis revealed severe lactic acidosis (pH, 6.78; lactate, 18 mmol/L). Blood tests showed leukocytosis, marked thrombocytopenia (platelet count: 5,000/µL), an elevated D-dimer level, and markedly elevated C-reactive protein and procalcitonin levels. The serum creatinine and blood urea nitrogen levels were also markedly elevated (5.91 and 114.5 mg/dL, respectively), indicating severe acute kidney injury compared with the creatinine level obtained eight days earlier, whereas the transaminase levels were within the reference ranges. Hypoalbuminemia (2.5 g/dL) and an elevated alkaline phosphatase level (649 U/L) were also noted. Her blood pressure subsequently decreased to 64/44 mmHg. Based on the clinical presentation and laboratory findings, sepsis with severe circulatory failure and multiorgan dysfunction was suspected. Sepsis-associated disseminated intravascular coagulation was also strongly suspected based on the marked thrombocytopenia and abnormalities of the coagulation and fibrinolytic systems (prothrombin time-international normalized ratio {PT-INR}, 1.28; D-dimer, 17.6 µg/mL).

**Table 1:** Laboratory data on admission Blood gas values, including glucose, were obtained from the first arterial blood gas analysis after arrival and the other values from the initial blood sample on arrival. Reference ranges are those of our institution's clinical laboratory PaCO₂, partial pressure of arterial carbon dioxide; HCO₃⁻, bicarbonate; PT-INR, prothrombin time-international normalized ratio; eGFR, estimated glomerular filtration rate

Parameter	Value on admission	Reference range
Arterial blood gas analysis		
pH	6.78	7.35-7.45
PaCO₂ (mmHg)	24.9	35-45
HCO₃⁻ (mmol/L)	3.5	22-26
Lactate (mmol/L)	18	0.5-1.6
Glucose (mg/dL)	75	65-95
Complete blood count		
White blood cell count (/µL)	23,600	3,300-8,600
Hemoglobin (g/dL)	13.1	11.6-14.8
Platelet count (/µL)	5,000	158,000-348,000
Coagulation and fibrinolysis		
PT-INR	1.28	0.88-1.09
Fibrinogen (mg/dL)	879	168-355
D-dimer (µg/mL)	17.6	<1.0
Blood chemistry		
Albumin (g/dL)	2.5	4.1-5.1
Total bilirubin (mg/dL)	0.7	0.4-1.5
Aspartate aminotransferase (U/L)	15	13-30
Alanine aminotransferase (U/L)	16	7-23
Alkaline phosphatase (U/L)	649	38-113
Lactate dehydrogenase (U/L)	322	124-222
Creatine kinase (U/L)	192	41-153
Amylase (U/L)	93	44-132
Blood urea nitrogen (mg/dL)	114.5	8.0-20.0
Creatinine (mg/dL)	5.91	0.46-0.79
eGFR (mL/minute/1.73 m²)	6.3	-
Sodium (mmol/L)	138	138-145
Potassium (mmol/L)	4.4	3.6-4.8
Chloride (mmol/L)	91	101-108
C-reactive protein (mg/dL)	41.72	0.00-0.30
Procalcitonin (ng/mL)	64.8	<0.05

Because of severe renal dysfunction, noncontrast CT of the chest, abdomen, and pelvis was performed on arrival (day 9). Compared with CT performed approximately six months earlier for postoperative follow-up of the pancreatic tumor, both kidneys and adrenal glands, as well as the liver, showed diffuse enlargement, accompanied by the increased attenuation of the perirenal and renal sinus fat (Figure 1).

**Figure 1:**
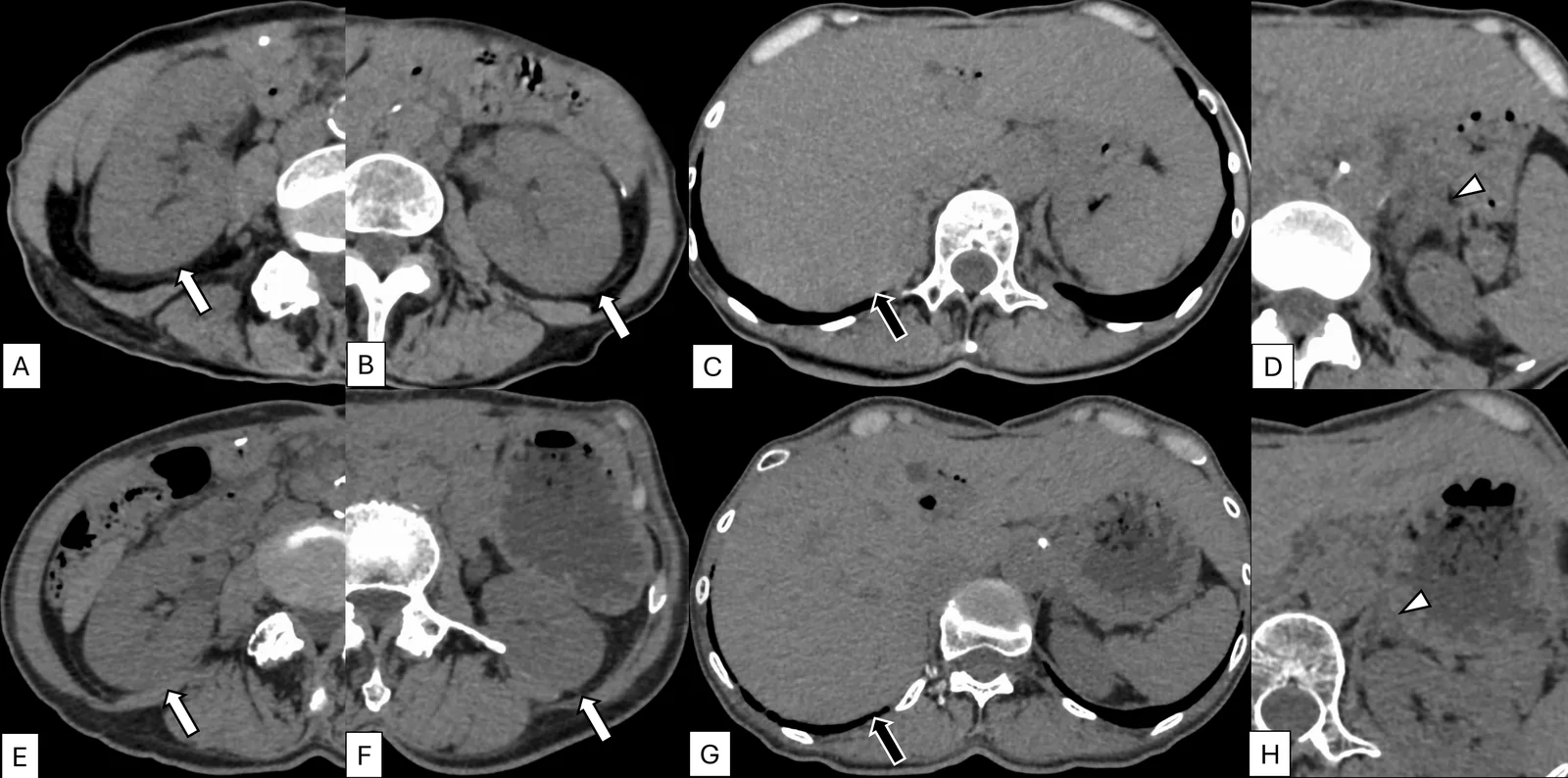
Abdominal computed tomography findings (A-D) Noncontrast abdominal computed tomography (CT) at the time of admission. (E-H) Noncontrast abdominal CT six months earlier. Comparison with the previous CT images demonstrates the enlargement of both kidneys (A, B, E, and F: white arrows; right kidney volume: 177 mL ← 147 mL; left kidney volume: 224 mL ← 161 mL), the liver (C and G: black arrows; volume: 1,404 mL ← 1,257 mL; CT value: 51 HU ← 62 HU), and both adrenal glands (D and H: arrowheads; right adrenal gland is not shown). Fat stranding in the perirenal and renal sinus regions is also observed

Both examinations were performed using our routine clinical abdominal CT protocol. Organ volumes were measured by manually tracing the organ contours on each axial slice, and hepatic attenuation was measured using a single large region of interest placed at comparable locations on both examinations, avoiding major vessels. The decrease in hepatic attenuation may be consistent with increased tissue water content; however, it should be interpreted with caution because it may also be affected by fatty change or minor differences in acquisition.

No definite abscess, gas within the organs, or focal fluid collection was identified. There was no bowel wall thickening, intestinal obstruction, enlargement of the remnant pancreas, main pancreatic duct dilatation, or significant lymphadenopathy. Based on the imaging findings, severe infection, including bilateral pyelonephritis, and its systemic effects were considered the primary possibility, with infiltrative diseases such as malignant lymphoma included in the differential diagnosis. The acute clinical course and marked inflammatory response were more suggestive of severe infection.

After two sets of blood cultures were obtained, empirical antimicrobial therapy with meropenem (2 g every eight hours) and vancomycin (0.75 g every 12 hours) was initiated on the day of admission. The patient received fluid resuscitation, continuous infusion of norepinephrine (0.9 mg/hour), mechanical ventilation, and continuous hemodiafiltration under intensive care. However, multiorgan failure progressed rapidly. On the day after admission (day 10), Gram-negative bacilli were detected in two sets of blood cultures and were ultimately identified as *E. tarda*. The isolate was susceptible to all antimicrobial agents tested, including ampicillin, cefazolin, ceftriaxone, meropenem, and levofloxacin, except for intermediate susceptibility to cefaclor. Further history-taking revealed that the patient had consumed eel on the day of symptom onset. Given the presence of diarrhea and vomiting, gastrointestinal entry was considered the most likely portal of entry; however, neither stool cultures nor the examination of the consumed food was performed. On day 10, laboratory tests showed marked elevations of AST (2,806 U/L), ALT (739 U/L), and creatine kinase (15,009 U/L). The patient died of multiorgan failure on day 10 (hospital day 2), the day after the CT examination. An autopsy was not performed.

## Discussion

This case has three important features. First, it was a fulminant case of *E. tarda* sepsis that progressed within a short period from symptom onset to severe lactic acidosis, disseminated intravascular coagulation (DIC), and multiorgan failure. Second, typical findings indicating localized infection, such as abscess formation or gas, were absent. Third, comparison with CT obtained approximately six months earlier demonstrated the enlargement of both kidneys and adrenal glands, as well as the liver. Although the interval between the two examinations precludes the definitive determination of the onset, these changes were highly likely to have developed acutely and nearly simultaneously, given the normal serum creatinine and transaminase levels eight days before admission and the fulminant clinical course.

The relationship between *E. tarda* and eels in Japan extends beyond dietary exposure to the history of the description of this bacterium and the eel-farming industry. In 1962, Hoshina described a bacterium isolated from diseased pond-cultured eels in Japan as a new species: *Paracolobactrum anguillimortiferum *[6]. Wakabayashi and Egusa subsequently showed that isolates from diseased cultured eels were identifiable as *E. tarda*, with which *Paracolobactrum anguillimortiferum* was considered synonymous [7]. Japan has a custom of consuming eel during the summer that is said to date back to the 18th century, and cases of *E. tarda* sepsis in patients with a history of consuming seafood, including eel, have been reported [8]. Therefore, the present case, in which the patient had consumed eel on the day of symptom onset, is of interest in light of the historical and cultural relationship between *E. tarda* and eels in Japan.

The mortality rate of *E. tarda* sepsis varies considerably depending on the study population and source of infection. In a review of 77 cases, mainly case reports, by Hirai et al., the overall mortality rate was 44.6%, and the mortality rate among cases with soft tissue infection was 61.1% [2]. In contrast, two recent single-center cohorts from Japan (26 and 37 patients, respectively) reported 30-day mortality rates of 12% and 8.6%, respectively, and 90-day mortality rates of 27% and 25.8%, respectively, with most late deaths attributable to underlying diseases such as advanced cancer rather than to the infection itself [3,4]. In the recent systematic review of 59 cases, 10 patients (16.9%) died [5]. Thus, mortality from *E. tarda* sepsis is considered to be influenced by underlying diseases, such as liver cirrhosis and malignancy, and the feasibility of source control. The present patient died on day 9 after symptom onset and can be regarded as having followed a fulminant course among cases of *E. tarda* infection.

Liver cirrhosis, malignancy, biliary tract disease, and diabetes mellitus have been reported as underlying factors associated with invasive *E. tarda* infection [2-5]. In the present case, pancreatogenic diabetes mellitus and a history of surgery for malignancies may have predisposed the patient to severe infection. In two recent Japanese single-center cohorts, hepatobiliary infections (cholangitis, cholecystitis, and liver abscess) accounted for the majority of sources (58% in one cohort and the large majority in the other), whereas enterocolitis was the source in only 15% and 0% of cases, respectively [3,4]. In the present case, CT showed no findings suggestive of biliary tract infection or pancreatitis of the remnant pancreas. Given the history of eel consumption and gastrointestinal symptoms, gastrointestinal entry was considered the most likely portal, although this remains presumptive.

Regarding the imaging findings of *E. tarda* sepsis, Suzuki et al. reported a patient with recurrent gastric cancer who had frequently eaten raw fish and grilled eel, in whom *E. tarda* was isolated from both blood and urine [8]; contrast-enhanced CT showed bilateral renal enlargement with perinephric stranding and bilateral psoas abscesses, and vertebral osteomyelitis (L2-L4) with a subsequent spinal epidural abscess was documented. Tominaga et al. reported an emphysematous liver abscess in an immunosuppressed woman, from which both *E. tarda* and *Escherichia coli *were isolated [9]. In these previously reported cases, localized lesions indicating a source of infection, such as pyelonephritis, abscess formation, or gas formation, were clearly demonstrated on imaging. Similarly, among the 32 patients who underwent CT in the recent systematic review, the reported findings mainly comprised localized lesions [5]. In contrast, the imaging feature of the present case was the enlargement of both kidneys and adrenal glands, as well as the liver, which was highly likely to have developed acutely and nearly simultaneously, in the absence of a localized abscess or gas.

The mechanism underlying the multiorgan enlargement in the present case remains unclear, and the following interpretation is speculative. In sepsis, endothelial injury and microcirculatory dysfunction can increase vascular permeability, resulting in the movement of intravascular fluid into the interstitial space and subsequent tissue edema [10]. In addition, adrenal gland volume has been reported to be greater in patients with septic shock than in control subjects, suggesting that adrenal enlargement may represent part of the systemic stress response [11]. Therefore, the enlargement of multiple organs observed in the present case may have reflected increased vascular permeability, interstitial edema, and hemodynamic changes associated with sepsis. Several alternative or contributory explanations should be considered. Renal enlargement with perirenal and renal sinus fat stranding may also reflect direct bacterial involvement, such as bilateral acute pyelonephritis, or sepsis-associated acute kidney injury itself. Hepatic enlargement may reflect venous congestion or ischemia-reperfusion injury after shock; however, the transaminase levels were within the reference ranges at the time of CT and rose markedly only on the following day, suggesting that the hepatic enlargement preceded overt ischemic hepatic injury. Adrenal enlargement may represent a physiological stress response to critical illness, whereas adrenal hemorrhage was considered less likely because no hyperattenuation was observed [11]. Fluid resuscitation around the time of CT may also have contributed to tissue edema. Infiltrative disease such as malignant lymphoma could not be completely excluded because no histopathological examination was performed, although the acute course, marked inflammatory response, and bacteremia favored a septic process.

This report has several limitations. First, because the comparison CT was obtained approximately six months earlier and no interim imaging was available, the onset and simultaneity of the organ enlargement could not be definitively established, although the normal serum creatinine and transaminase levels eight days before admission and the fulminant course strongly suggest that these changes developed acutely. Second, the source of infection was presumptive; neither stool cultures nor the examination of the consumed food was performed, and eel consumption on the day of symptom onset does not establish causality. Third, no autopsy was performed, and the histopathological correlation of the organ enlargement was therefore unavailable. Finally, observations from a single case cannot establish a causal relationship between *E. tarda* sepsis and this imaging pattern.

## Conclusions

We report a fatal case of *E. tarda* bacteremia and sepsis in a patient with recent eel consumption, in whom comparison with CT obtained approximately six months earlier demonstrated the enlargement of both kidneys and adrenal glands, as well as the liver, which was highly likely to have developed acutely. Although the source of infection could not be definitively established, this case suggests that severe *E. tarda* sepsis may be associated with the diffuse enlargement of multiple abdominal organs and progress rapidly to multiorgan failure without localized imaging findings such as liver abscesses or gas-forming lesions. When no apparent focal source of infection is identified despite a clinical presentation suggestive of severe infection, prompt blood culture testing and comparison with previous imaging studies are important, and a history of seafood or freshwater fish consumption, including eel, may serve as a supportive clue rather than a prerequisite for considering *E. tarda* infection.
